# Phloridzin mitigates bleomycin-elicited lung fibrosis in Wistar rats: The interplay between antioxidant defenses, inflammatory processes, transforming growth factor beta 1, and autophagy

**DOI:** 10.1016/j.toxrep.2026.102267

**Published:** 2026-05-03

**Authors:** Naif M.F. Alenezi, Abeer Elkhoely, Ahmed M. Kabel, Amany A.E. Ahmed

**Affiliations:** a Ministry of Health, Northern Border Health Cluster, Ar’ar, Kingdom of Saudi Arabia; b Pharmacology and Toxicology Department, Faculty of Pharmacy, Helwan University, Cairo, Egypt; c Pharmacology Department, Faculty of Medicine, Tanta University, Tanta, Egypt

**Keywords:** phloridzin, bleomycin, pulmonary fibrosis, oxidative stress, inflammation, rats

## Abstract

Pulmonary fibrosis is a serious complication that limits the clinical use of bleomycin (BLM). This study investigated the protective role of phloridzin (PZ) against BLM-induced pulmonary fibrosis in rats. In a rat model of BLM-elicited lung fibrosis, PZ was administered daily at two dose levels for 35 days, beginning 7 days before BLM treatment. PZ significantly reduced the elevated total leukocyte count, neutrophil and lymphocyte percentages, and lactate dehydrogenase (LDH) activity in the bronchoalveolar lavage fluid (BALF), while increasing BALF macrophage levels. It also lowered malondialdehyde and increased glutathione concentrations. Furthermore, PZ pre-treatment suppressed transforming growth factor-beta 1, interleukin-1β, and nuclear factor-kappa B, while reducing cleaved caspase-3 expression and restoring beclin-1 tissue levels. Histopathological analysis confirmed these protective effects. Taken together, PZ mitigated oxidative stress, inflammation, and apoptosis, while supporting autophagic activity, thereby demonstrating a strong protective effect against BLM-induced lung fibrosis. Importantly, this work provides the first evidence that PZ may counteract multiple pathological mechanisms of BLM-elicited lung toxicity, positioning it as a promising candidate for therapeutic intervention in drug-induced pulmonary fibrosis.

## Highlights

• The effect of phloridzin on bleomycin (BLM)-induced lung toxicity was assessed.
• Phloridzin dose-dependently ameliorated BLM effects on leucocytic count.
• Phloridzin dose-dependently attenuated oxidative stress & inflammation.
• Phloridzin dose-dependently restored autophagy/apoptosis balance.
• Phloridzin dose-dependently combated BLM-induced fibrotic changes in the lung.

## Graphical Abstract

**Figure:**
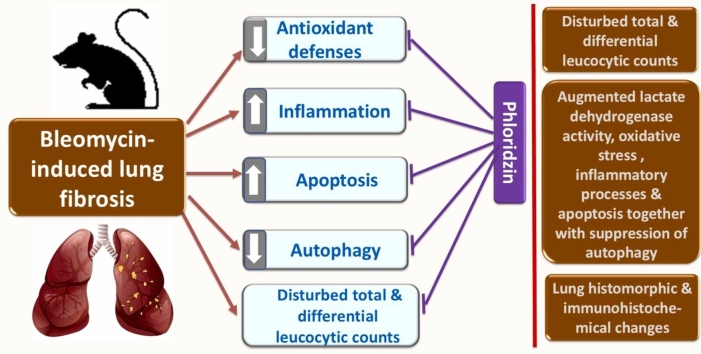

## 1 Introduction

Pulmonary fibrosis (PF) refers to a group of chronic lung conditions that cause progressive scarring of the pulmonary tissue and lead to impaired respiratory functions [1]. Until now, no single factor can be considered to be the main cause of PF [2]. However, genetic predisposition, autoimmune disorders, environmental exposures, and certain drugs have all been implicated [3]. Most cases are idiopathic, with disease mechanisms involving epithelial cell injury, activation of transforming growth factor beta-1 (TGF-β1), oxidative stress, and dysregulated apoptosis contributing to fibrotic remodeling of the lung [4].

Until now, no single therapeutic modality can be considered to offer a complete cure of PF [5]. Despite advances, PF remains incurable. Current antifibrotic therapies—pirfenidone and nintedanib—slow disease progression and improve survival, but their use is limited by cost and adverse effects [6], [7]. As a consequence, the ongoing research tries to explore new effective and safe treatments for the amelioration of the pathogenic events of PF [8].

Bleomycin, a chemotherapeutic agent, is well known to induce PF as a serious adverse effect [9]. Its pulmonary toxicity is linked to oxidative stress, inflammation, and disruption of autophagy/apoptosis balance [10]. This results in excessive extracellular matrix deposition with subsequent derangement of the pulmonary tissue architecture with the end result of respiratory failure [11].

Natural compounds with antioxidant and anti-inflammatory properties are being investigated as potential protective agents against BLM-induced fibrotic changes in the lung [12]. Phloridzin is one of the flavonoids that belongs to the chemical class of dihydrochalcones. It is found in considerable amounts in apple leaves and bark, but also in smaller amounts in fruits [13]. It is a glucoside of phloretin that inhibits renal glucose transport with subsequent reduction of elevated blood glucose levels [14]. Interestingly, recent reports highlighted the potent antioxidant effects of phloridzin, possibly mediated via interference with reactive oxygen species (ROS) production and augmentation of the antioxidant defense mechanisms [15]. These effects together with its unique ability to mitigate the inflammatory responses and restore autophagy/apoptosis balance, may suggest a role for phloridzin in the amelioration of the pathological states in which oxidative stress, inflammatory events, and autophagy play a pathogenic role [16]. Based on its pharmacological profile, we hypothesized that PZ could counteract BLM-induced lung fibrosis by reducing oxidative stress, suppressing inflammation and apoptosis, and restoring autophagic balance. This study is the first to investigate PZ in the context of pulmonary fibrosis, aiming to establish its protective efficacy and uncover its underlying mechanisms. By addressing this unexplored therapeutic avenue, the current work introduces PZ as a promising candidate for mitigating drug-induced pulmonary toxicity.

## 2 Materials and methods

### 2.1 Drugs and chemicals

Bleomycin, a white powder with a purity of ≥ 98%, a product of Celon Laboratories Ltd., Hyderabad, Telangana, India (CAS # 9041–93–4). Phloridzin, a powder form with a purity of 99%, was obtained from Shanghai Nianxing Industrial Co., Shanghai, China (CAS # 7061–54–3). All other chemicals and reagents used were of high analytical grade and were obtained from The Egyptian Company for Biotechnology, Cairo, Egypt.

### 2.2 Animals

The handling of animals as well as the experimental procedures were performed in the Medical Pharmacology Department, Faculty of Medicine, Tanta University, Egypt, in accordance with the ARRIVE guidelines for animal experiments. The protocol of the present study was approved by the Research Ethics Committee, Faculty of Pharmacy, Helwan University, Egypt (15A2024). The present study was conducted on 75 male Wistar rats obtained from a local source, weighing between 150 and 200 g and aged 8–10 weeks. Rats were housed in wire mesh cages with the appropriate hygienic measures. They had free access to food and water *ad libitum* throughout the whole duration of the experiment.

### 2.3 Experimental design

Rats were allowed to acclimatize for one week and then were randomly divided into five equal groups (15 rats for each) as follows: Group 1 (Vehicle control group) in which rats received 5% DMSO solution daily by oral gavage starting from the first day of the experiments for 35 days, and on the 8th day, they were given normal saline intranasally using a micropipette once daily for 6 days; Group 2 (phloridzin control group) in which rats received phloridzin in a dose of 120 mg/kg [17] dissolved in 5% DMSO solution daily by oral gavage starting from the first day of the experiments for 35 days, and on the 8th day, they were given normal saline intranasally using a micropipette once daily for 6 days; Group 3 in which rats received bleomycin intranasally using a micropipette (0.25 mg/kg/day) once daily starting from the 8th day of the experiments for 6 days [18]; and Groups 4 and 5 in which rats were pre-treated daily orally with phloridzin at a daily dose of 60 and 120 mg/kg, respectively, by oral gavage starting from the first day of the experiment for 35 days, and on the 8th day, they were given bleomycin intranasally using a micropipette (0.25 mg/kg/day) once daily for 6 days [17].

### 2.4 Broncho-alveolar lavage fluid (BALF) collection and tissue sampling

At the end of the experiment (the 36th day), rats were fasted overnight, then euthanized with intraperitoneal injection of a high dose of thiopental (70 mg/kg) [19], and the lungs were exposed. The main bronchus of the left lung was ligated by silk suture and a 14-G cannula was inserted into the main bronchus of the right lung. Then, the right lung was lavaged with 6 mL ice-cold saline for 4 times [20]. BALF was centrifuged at 3000 rpm for 10 min. Then, the supernatants were harvested and utilized for the assessment of lactate dehydrogenase (LDH) activity. The cell pellets were re-suspended in 1 mL saline and used for quantification of the total and differential leucocytic counts. After that, the lungs were dissected. Parts of the left lung were processed for histopathological and immunohistochemical examination. The remaining parts were homogenized using Acculab USA homogenizer, and the resulting homogenate was centrifuged for 15 min at 3000 rpm at 4°C. Then, the samples were placed on ice and the supernatant was added to a sterile Eppendorf tube and stored at −80°C to be processed for further assay of the biochemical parameters. In the present study, all experiments were performed with three biological replicates per group, and each measurement was repeated in triplicate technical replicates to ensure reliability and reproducibility of the data.

### 2.5 Quantification of the total and differential leucocytic counts in BALF

The total and differential leucocytic counts in BALF were quantified by hemocytometer LHCT-A10 (Labtron Equipment Ltd., Camberley, Surrey GU16 7ER, United Kingdom) after staining with Turk solution, which was prepared as a mixture of 3 mL glacial acetic acid, 1 mL gentian violet 1% and then made to 100 mL by distilled water [21].

### 2.6 Quantification of lactate dehydrogenase (LDH) activity in BALF

BALF LDH activity in of the studied groups was determined using colorimetric kits supplied by The Egyptian Company for Biotechnology, Cairo, Egypt (Catalog number 283 001) according to the supplier's guide.

### 2.7 Assessment of the lung tissue biochemical parameters

MDA and GSH levels were estimated using kits supplied by Biodiagnostic, Giza, Egypt (Catalog number MD 25–29) and Beijing Solarbio Science & Technology Co., Ltd., Beijing, China (Catalog number BC1170), respectively. Using ELISA kits supplied by DLDEVELOP, China, the following parameters were assessed: IL-1β (Catalog number DL-IL1β-Ra), NF-κB (Catalog number DL-NFκB-Ra) and TGF-β1(Catalog number DLR-TGFb1-Ra). The afore-mentioned biochemical parameters in lung tissue samples were assessed using a quantitative sandwich ELISA technique. In this method, microplates were coated with antibodies specific to the target analyte. Standards or samples were added to the wells, followed by a biotin-labeled antibody against the same analyte. Subsequently, horseradish peroxidase (HRP)–conjugated avidin was added and incubated. The reaction was then developed with a TMB substrate, producing a color change in the wells containing the analyte–antibody complexes. The resulting color intensity was measured spectrophotometrically at the designated wavelength.

### 2.8 Immunohistochemical analysis of the harvested lung tissue specimens

The immunohistochemical expression of cleaved caspase-3 and beclin-1 was determined in the harvested lung tissue specimens after formalin fixation and paraffin-embedding according to Buyuklu et al. method [22] and Wasfey et al. method [23], respectively. The formalin-fixed paraffin-embedded slices were cut into thin 4, 5 μm thickness) by Leica ultramicrotome (Leica, Wetzlar, Hesse, Germany). After that, the tissue samples were deparaffinized with xylene and subsequently rehydrated through a graded ethanol series. Antigen retrieval was carried out in citrate buffer (pH 6.0) using microwave heating to expose the epitopes. Endogenous peroxidase activity was quenched by treating the thin tissue sections with hydrogen peroxide, followed by the application of blocking serum to prevent non‑specific binding. The sections were then incubated overnight at 4 °C with a rabbit monoclonal primary antibody against cleaved caspase‑3 (Cell Signaling Technology, Danvers, MA, USA; catalog no. 9664) and a primary rabbit beclin-1 polyclonal antibody obtained from Elabscience, United States (Catalog number E-AB-53242). After rinsing, a biotinylated secondary antibody (MyBioSource, San Diego, CA, USA; catalog no. MBS9610384) was applied to bind the primary antibody for cleaved caspase-3 and a secondary antibody conjugated to horseradish peroxidase enzyme (Santa Cruz Biotechnology, Inc., Dallas, Texas, United States, catalog number sc-48381) was applied to bind the primary antibody for beclin-1. Visualization was achieved using the avidin–biotin complex method for cleaved caspase-3 and chromogenic substrate (DAB) which produces a brown precipitate at the site of beclin-1 expression. Hematoxylin was used as a counterstain to highlight nuclei, after which the slides were dehydrated through ascending ethanol concentrations, cleared in xylene, and mounted with Permount medium and coverslips. A Leica DM2000 light microscope (Leica Microscopy and Scientific Instruments Group, Germany) was employed to evaluate staining intensity in ten randomly selected high‑power fields (×400) of lung tissue. The immunohistochemical staining was classified as negative (0, no immunoreactive cells), mild (+, <5% positive cells), moderate (++, 5–50% positive cells), or strong (+++, >50% positive cells). Quantification of cleaved caspase‑3 and beclin-1 expression was performed using ImageJ software (version 1.52 f, NIH, Bethesda, MD, USA).

### 2.9 Histopathological examination of the harvested lung tissue specimens

Sections of the left lung were preserved in 10% formalin for 48 h, dehydrated with graded ethyl alcohol, and embedded in paraffin to produce tissue blocks. These blocks were sectioned at 5 μm thickness using a microtome, mounted on glass slides, and stained with hematoxylin and eosin (H&E) for histopathological evaluation under an Olympus light microscope (Olympus, Tokyo, Japan). In addition, Masson’s trichrome staining was performed to evaluate collagen fiber deposition within the lung tissue. The extent of collagen accumulation was quantified microscopically (Olympus, Tokyo, Japan) using ImageJ software (NIH, Bethesda, MD, USA), and fibrosis scoring was carried out according to established protocols from previous studies [24]. The fibrotic alterations in lung tissue were assessed using a nine‑point grading system in which grade 0 denotes normal lung histology with no fibrotic changes; grade 1 refers to minimal fibrous alterations confined to isolated inter‑alveolar septa; grade 2 explains knot‑like fibrotic structures appearing within the inter‑alveolar septa; grade 3 denotes contiguous fibrotic thickening of the inter‑alveolar septal walls; grade 4 refers to scattered fibrotic aggregates distributed throughout the lung parenchyma; grade 5 means confluent fibrotic masses clearly visible in the lung tissues; grade 6 demonstrates extensive, large, and continuous fibrotic masses within pulmonary tissue; grade 7 refers to the presence of air bubbles detected in the lung specimens, and grade 8 demonstrates complete obliteration of the airways by fibrous tissue. Each lung section was examined under light microscopy, and scores were assigned according to the defined criteria (grades 0–8). To ensure reliability, evaluations were performed independently by two experienced pathologists who were blinded to the experimental groups. For each animal, the fibrosis score was expressed as the mean value obtained from multiple sections. Inter‑observer consistency was checked, and any differences were resolved through joint discussion. This procedure ensured both reproducibility and validation of the scoring system in the present study.

### 2.10 Statistical evaluation

The obtained information from the results was tabulated and statistically analyzed using GraphPad Prism 9 for Windows. The Shapiro-Wilk test for assessment of normality was performed. The parametric values were represented as mean ± standard deviation (SD) and compared by one-way ANOVA and post-hoc Tukey's multiple comparison test, while the non-parametric values were expressed as median (interquartile range, IQR) and compared by Kruskal-Wallis and Mann-Whitney U tests. The F-value was calculated, and the p-value was determined. Values of p < 0.05 were considered significant.

## 3 Results

### 3.1 Effect of bleomycin with or without phloridzin on BALF total and differential leucocytic counts

Table 1 shows the impact of PZ on leucocytic count. The group treated with PZ alone didn't exhibit a significant effect on the BALF total leucocytic count when compared to the control group (p > 0.05). Administration of bleomycin (0.25 mg/kg/day, intranasal once daily for 6 days) induced a significant increase in the total leucocytic count in BALF by 7.7-fold as compared to the control group. Nevertheless, treatment with phloridzin (60 and 120 mg/kg/day, p.o.) showed a significant decrease in the total leucocytic count in BALF by 23.1% and 52.8% respectively, compared to the bleomycin group. Moreover, phloridzin (120 mg/kg/day) showed a remarkable decrease in the total leucocytic count in BALF by 38.6% compared to the bleomycin group treated by phloridzin (60 mg/kg/day).

**Table 1:** Effect of bleomycin with or without phloridzin on BALF total and differential leucocytic counts.

	Control	PZ120	BLM	BLM + PZ60	BLM + PZ120
Total leucocytic count (×10^4^)	39.8 ± 5.89	42.3 ± 6.02	305.48 ± 32.39^a^	234.77 ± 25.17^a,b^	144.06 ± 18.83^a,b,c^
Macrophages (%)	80.19 ± 8.25	82.31 ± 8.33	39.29 ± 5.13^a^	54.82 ± 5.15^a,b^	69.43 ± 7.28^a,b,c^
Neutrophils (%)	1.85 ± 0.33	1.93 ± 0.35	5.71 ± 0.69^a^	4.24 ± 0.38^a,b^	3.23 ± 0.41^a,b,c^
Lymphocytes (%)	11.24 ± 2.15	12.36 ± 2.29	52.23 ± 4.19^a^	34.54 ± 4.74^a,b^	20.16 ± 2.9^a,b,c^

The group treated with PZ alone didn't show a significant effect on the percentage of neutrophils, macrophages, and lymphocytes in BALF when compared to the control group (p > 0.05). Administration of bleomycin (0.25 mg/kg/day, intranasal once daily for 6 days) induced a significant increase in the percentage of neutrophils and lymphocytes in BALF by 3.08-fold and 4.6-fold respectively, associated with a significant decline in the percentage of macrophages in BALF by 50.9% as compared to the control group. Nevertheless, treatment with phloridzin (60 mg/kg/day, p.o.) showed a significant decrease in the percentage of neutrophils and lymphocytes in BALF by 25.6% and 33.9% respectively associated with a substantial increase in the percentage of macrophages in BALF by 39.5% as compared to the bleomycin group. Also, treatment with phloridzin (120 mg/kg/day, p.o.) showed a significant decrease in the percentage of neutrophils and lymphocytes in BALF by 43.4% and 61.4% respectively, associated with a significant increase in the percentage of macrophages in BALF by 76.7% as compared to the bleomycin group. Moreover, phloridzin (120 mg/kg/day) showed a remarkable decrease in the percentage of neutrophils and lymphocytes in BALF by 23.9% and 41.6% respectively, associated with a significant increase in the percentage of macrophages in BALF by 26.6% compared to the bleomycin group treated by phloridzin (60 mg/kg/day) (Table 1).

Values are expressed as mean ± standard deviation. Number of animals = 15 rats in each group. One-way analysis of variance (ANOVA) was used to assess the differences between the different groups, and then Tukey's multiple comparisons post-hoc test was employed. A *p-value* less than 0.05 was considered significant. ^a^ significantly different from the control group; ^b^ significantly different from the untreated BLM group; ^c^ significantly different from the BLM+PZ60 group. BLM: bleomycin; PZ: phloridzin.

### 3.2 Effect of bleomycin with or without phloridzin on BALF LDH activity

**Fig. 1:**
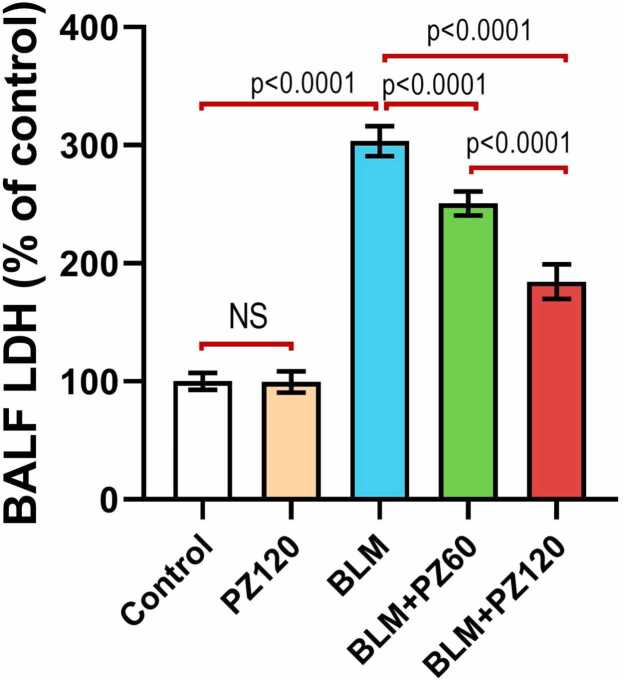
Phloridzin dose-dependently mitigated the effect of bleomycin on BALF LDH activity. Values are expressed as mean ± standard deviation. Number of animals = 15 rats in each group. One-way analysis of variance (ANOVA) was used to assess the differences between the different groups, and then Tukey's multiple comparisons post-hoc test was employed. A *p-value* less than 0.05 was considered statistically significant. BLM: bleomycin; LDH: lactate dehydrogenase; NS: non-significant; PZ: phloridzin.

As depicted in Fig. 1, administration of PZ alone didn't produce a significant effect on BALF LDH activity relative to the control group (p > 0.05). Bleomycin (0.25 mg/kg/day, intranasal once daily for 6 days) induced a significant increase in BALF LDH activity by 3.04-fold as compared to the control group. Nevertheless, treatment with phloridzin (60 and 120 mg/kg/day, p.o.) showed a significant decrease in BALF LDH activity by 17.4% and 39.2% respectively, compared to the bleomycin group. Moreover, phloridzin (120 mg/kg/day) showed a remarkable decrease in BALF LDH activity by 26.4% compared to the bleomycin group treated by phloridzin (60 mg/kg/day).

### 3.3 Effect of bleomycin with or without phloridzin on oxidative stress markers in the lung tissues

**Fig. 2:**
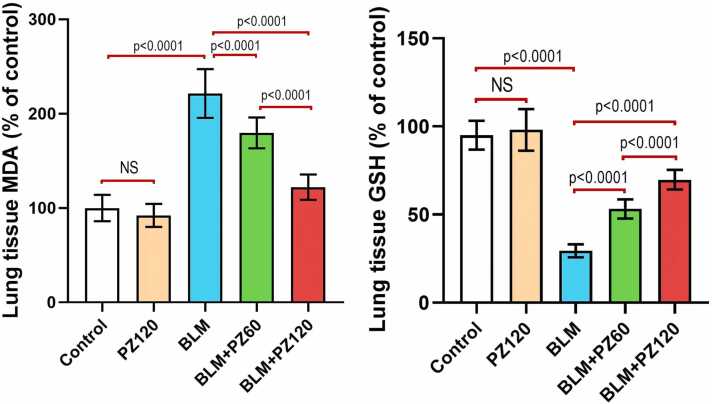
Phloridzin dose-dependently exhibited antioxidant effects in bleomycin-treated animals. Values are expressed as mean ± standard deviation. Number of animals = 15 rats in each group. One-way analysis of variance (ANOVA) was used to assess the differences between the different groups, and then Tukey's multiple comparisons post-hoc test was employed. A *p-value* less than 0.05 was considered statistically significant. BLM: bleomycin; GSH: reduced glutathione; MDA: malondialdehyde; NS: non-significant; PZ: phloridzin.

Fig. 2 elucidates the antioxidant effect of phloridzin in the lung of BLM-treated rats. Rats treated with PZ alone didn't show a significant effect on the oxidative stress markers in the lung tissues when compared to the control rats (p > 0.05). Administration of bleomycin (0.25 mg/kg/day, intranasal once daily for 6 days) induced a significant increase in the lung tissue MDA content by 2.2-fold as compared to the control group. Nevertheless, treatment with phloridzin (60 and 120 mg/kg/day, p.o.) showed a significant decrease in the lung tissue MDA content by 18.9% and 44.9%, respectively, compared to the bleomycin group. Moreover, phloridzin (120 mg/kg/day) showed a remarkable decrease in the lung tissue MDA content by 32.04% compared to the group treated with phloridzin (60 mg/kg/day). In addition, bleomycin (0.25 mg/kg/day, intranasal once daily for 6 days) induced a significant decrease in the lung tissue GSH level by 68.2% as compared to the control group. Contrarily, treatment with phloridzin (60 and 120 mg/kg/day, p.o.) showed a significant increase in the lung tissue GSH level by 80.9% and 137.3% respectively, compared to the bleomycin group. Moreover, phloridzin (120 mg/kg/day) showed a remarkable increase in the lung tissue GSH level by 31.2% compared to the bleomycin group treated by phloridzin (60 mg/kg/day).

### 3.4 Effect of bleomycin with or without phloridzin on the inflammatory parameters in the lung tissues

**Fig. 3:**
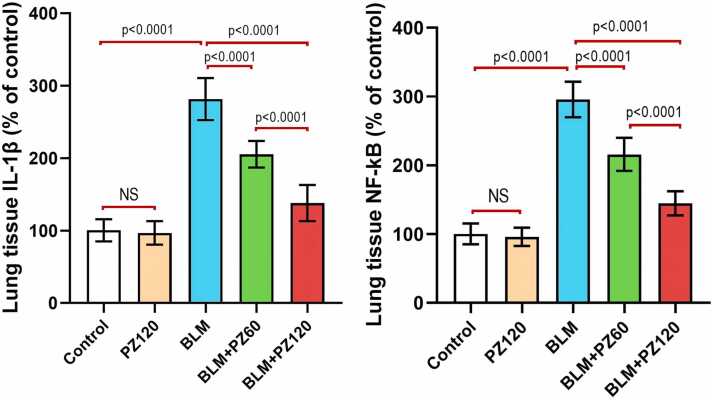
Phloridzin dose-dependently abrogated the effect of bleomycin on the inflammatory markers in the lung tissues. Values are expressed as mean ± standard deviation. Number of animals = 15 rats in each group. One-way analysis of variance (ANOVA) was used to assess the differences between the different groups, and then Tukey's multiple comparisons post-hoc test was employed. A *p-value* less than 0.05 was considered statistically significant. BLM: bleomycin; IL-1β: interleukin-1 beta; NF-κB: nuclear factor kappa B; NS: non-significant; PZ: phloridzin.

As shown in Fig. 3, administration of PZ alone didn't have a significant impact on the lung tissue IL-1β and NF-κB levels when compared to the control values (p > 0.05). Treatment with bleomycin alone (0.25 mg/kg/day, intranasal once daily for 6 days) induced a significant increase in the lung tissue IL-1β and NF-κB levels by 2.8- and 2.96-fold, respectively, as compared to the control group. Conversely, treatment with phloridzin (60 and 120 mg/kg/day, p.o.) showed a significant decrease in the lung tissue IL-1β level by 27.1% and 48.6%, respectively, and NF-κB level by 27.01% and 51.1%, respectively, compared to the bleomycin group. Moreover, phloridzin (120 mg/kg/day) showed a remarkable decrease in the lung tissue IL 1β and NF-κB levels by 29.6% and 32.97%, respectively compared to the bleomycin group treated by phloridzin (60 mg/kg/day)

### 3.5 Effect of bleomycin with or without phloridzin on TGF-β1 in the lung tissues

**Fig. 4:**
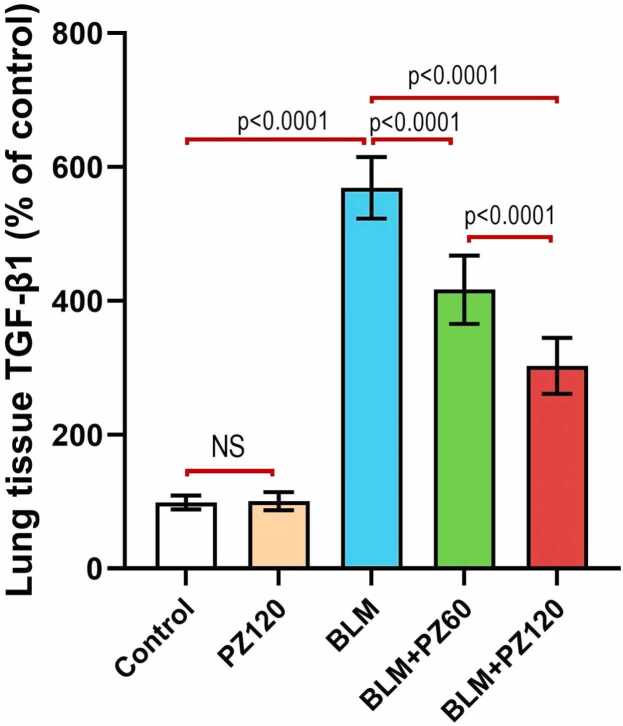
Phloridzin dose-dependently ameliorated the effect of bleomycin on TGF-β1 levels in the lung tissues. Values are expressed as mean ± standard deviation. Number of animals = 15 rats in each group. One-way analysis of variance (ANOVA) was used to assess the differences between the different groups, and then Tukey's multiple comparisons post-hoc test was employed. A *p-value* less than 0.05 was considered statistically significant. BLM: bleomycin; NS: non-significant; PZ: phloridzin; TGF-β1: transforming growth factor beta 1.

Animals treated with PZ alone didn't show a significant effect on the lung tissue TGF-β1 levels when compared to the control animals (p > 0.05). Administration of bleomycin (0.25 mg/kg/day, intranasal once daily for 6 days) induced a significant increase in the lung tissue TGF-β1 levels by 5.7-fold as compared to the control group. Contrarily, treatment with phloridzin (60 and 120 mg/kg/day, p.o.) showed a significant decrease in the lung tissue TGF-β1 levels by 26.7% and 46.8%, respectively, compared to the bleomycin group. Moreover, treatment with phloridzin (120 mg/kg/day) showed a remarkable decrease in the lung tissue TGF-β1 levels by 27.4% compared to the bleomycin group treated by phloridzin (60 mg/kg/day) (Fig. 4).

### 3.6 Effect of bleomycin with or without phloridzin on cleaved caspase-3 and beclin-1 expression in the lung tissues

**Fig. 5:**
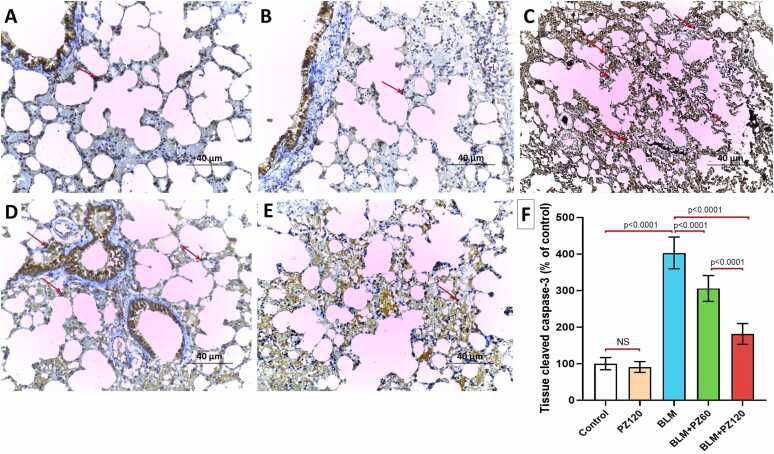
A photomicrograph demonstrating the immunohistochemical staining of the lung tissue specimens for cleaved caspase-3 (x200, scale bar=40 µm) from A) and B) the control group and PZ-alone treated group respectively showing minimal positive immune expression of cleaved caspase-3 (Arrow); C) the group treated with bleomycin alone showing strong positive immune expression of cleaved caspase-3 (Arrows); D) the bleomycin group treated with phloridzin 60 mg/kg/day showing moderate positive immune expression of cleaved caspase-3 (Arrows); E) the bleomycin group treated with phloridzin 120 mg/kg/day showing mild positive immune expression of cleaved caspase-3 (Arrows); and F) The percentage effect of phloridzin (60 and 120 mg/kg/day, p.o) on the immune expression of cleaved caspase-3 in the lung tissues of bleomycin-treated rats. Values are expressed as mean ± standard deviation. Number of animals = 15 rats in each group. One-way analysis of variance (ANOVA) was used to assess the differences between the different groups, and then Tukey's multiple comparisons post-hoc test was employed. A *p-value* less than 0.05 was considered statistically significant. BLM: bleomycin; NS: non-significant; PZ: phloridzin.

**Fig. 6:**
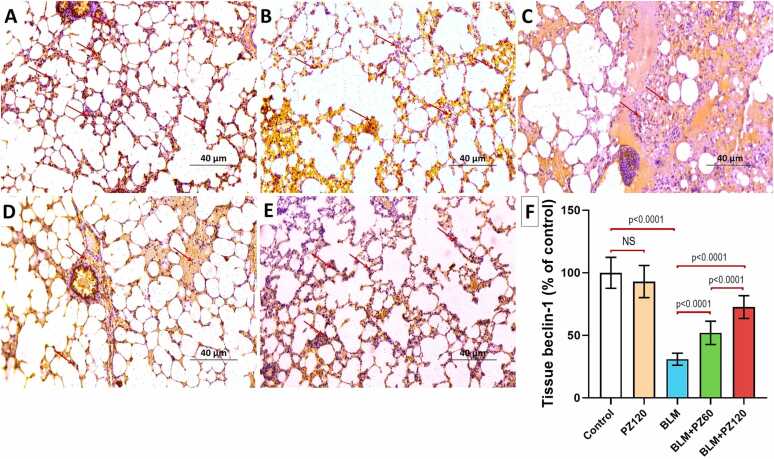
A photomicrograph demonstrating the immunohistochemical staining of the lung tissue specimens for beclin-1 (×200, scale bar = 40 µm) from A) and B) the control group and PZ-alone treated group respectively showing strong positive immune expression of beclin-1 (Arrows); C) the group treated with bleomycin alone showing minimal positive immune expression of beclin-1 (Arrows); D) the bleomycin group treated with phloridzin 60 mg/kg/day showing moderate positive immune expression of beclin-1 (Arrows); E) the bleomycin group treated with phloridzin 120 mg/kg/day showing strong positive immune expression of beclin-1 (Arrows); and F) The percentage effect of phloridzin (60 and 120 mg/kg/day, p.o) on the immune expression of beclin-1 in the lung tissues of bleomycin-treated rats. Values are expressed as mean ± standard deviation. Number of animals = 15 rats in each group. One-way analysis of variance (ANOVA) was used to assess the differences between the different groups, and then Tukey's multiple comparisons post-hoc test was employed. A *p-value* less than 0.05 was considered statistically significant. BLM: bleomycin; NS: non-significant; PZ: phloridzin.

As shown in Figs. 5A and 6A, the control group exhibited minimal immune expression and a strong positive immune expression of cleaved caspase-3 and beclin-1, respectively. Animals treated with PZ alone didn't show a significant effect on the lung tissue immune expression of cleaved caspase-3 and beclin-1 when compared to the control animals (Figs. 5B and 6B, respectively). Administration of bleomycin (0.25 mg/kg/day, i.n.) starting from the 8th day of the experiment for 6 days induced a significant increase and a remarkable decrease in the immune expression of cleaved caspase-3 and beclin-1, respectively, in the lung tissues relative to the control group (Figs. 5C and 6C). In contrast, treatment with phloridzin (60 and 120 mg/kg/day, p.o.) induced a dose-dependent significant decrease in the immune expression of cleaved caspase-3 (Fig. 5D-F) and a dose-dependent significant elevation in the immune expression of beclin-1 (Fig. 6D-F) in the lung tissues compared to the group treated with bleomycin alone.

### 3.7 Effect of bleomycin with or without phloridzin on the histopathological picture of the lung tissues

**Fig. 7:**
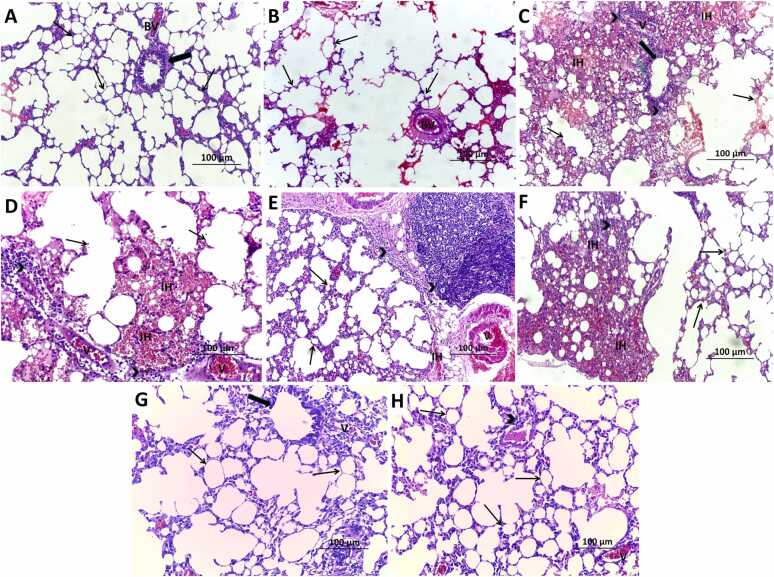
A photomicrograph of hematoxylin and eosin-stained sections (×100, scale bar = 100 µm) from the lung tissues of A) and B) the control group and PZ-alone treated group respectively showing normal lung morphological structure with intact alveoli (Thin arrows) and bronchioles (Thick arrows) with normal blood vessels (BV); C) and D) the group treated with bleomycin alone showing significant perturbation of the lung architecture as evidenced by destruction of the interalveolar septa (Thin arrows), distortion of the walls of the bronchi (Thick arrow), perinuclear vacuolation with pyknotic nuclei in the epithelial lining of the bronchioles (wavy green arrows), massive inflammatory cellular infiltration (Arrow heads), severe congestion of the vascular bed (V), with massive interstitial hemorrhage (IH); E) and F) bleomycin group treated with phloridzin (60 mg/kg/day) showing significant decline in the alveolar walls' destruction (Thin arrows), moderate inflammatory cellular infiltration (Arrow head), moderate vascular congestion (V), and significant decrease in the interstitial haemorrhage (IH); G) and H) bleomycin group treated with phloridzin (120 mg/kg/day) showing apparently normal alveoli (Thin arrows) with significant diminution of the inflammatory cellular infiltration (Arrow head) and minimal vascular congestion (V) with restoration of the bronchiolar wall (Thick arrow).

As elucidated in Fig. 7A, the lung tissues in the control group exhibited normal histological features with intact bronchioles, alveoli, and interalveolar septa. Administration of PZ alone didn't significantly affect the histopathological picture of the lung tissue relative to the control animals (Fig. 7B). Administration of bleomycin (0.25 mg/kg/day, intranasally) from day 8 of the experiment for six consecutive days resulted in marked architectural disruption, characterized by extensive alveolar wall damage, pronounced interstitial inflammatory cell infiltration, and severe vascular congestion (Fig. 7C and D). In contrast, treatment with PZ (60 and 120 mg/kg/day, orally) produced a dose‑dependent reduction in inflammatory infiltration and vascular congestion, leading to restoration of pulmonary tissue morphology (Fig. 7E-H).

**Fig. 8:**
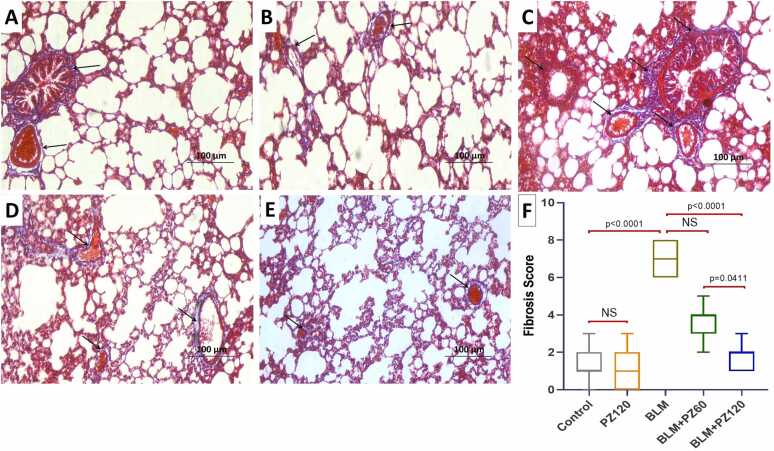
A photomicrograph of Masson’s trichrome stained sections (×200, scale bar = 100 µm) from the lung tissues of A) and B) the control group and PZ-alone treated group respectively showing minimal collagen fibers deposits around the bronchioles and the pulmonary blood vessels (Arrows); C) the group treated with bleomycin alone showing massive deposition of the collagen fibers around the alveoli, bronchioles, and pulmonary blood vessels in addition to the interalveolar septa (Arrows); D) bleomycin group treated with phloridzin (60 mg/kg/day) exhibiting a significant decline in the deposition of the collagen fibers around the pulmonary blood vessels and the alveoli, and in the interalveolar septa (Arrows); E) bleomycin group treated with phloridzin (120 mg/kg/day) exhibiting minimal deposition of the collagen fibers around the pulmonary blood vessels and the alveoli, in addition to the interalveolar septa (Arrows), and F) The effect of phloridzin (60 and 120 mg/kg/day, p.o) on the fibrosis score in the lung tissues harvested from bleomycin-treated rats. Values were expressed as median (Interquartile range, IQR) and compared using Kruskal-Wallis followed by Dunn's test. Number of animals = 15 rats in each group. A *p-value* less than 0.05 was considered statistically significant. BLM: bleomycin; NS: non-significant; PZ: phloridzin.

As illustrated in Figs. 8A and 8B, collagen fiber deposition around bronchioles and pulmonary vessels was minimal in the control and PZ-alone treated groups, respectively. Bleomycin exposure, however, significantly increased collagen accumulation around bronchioles, blood vessels, alveoli, and interalveolar septa compared with controls (Fig. 8C). Conversely, PZ treatment (60 and 120 mg/kg/day, orally) markedly and dose‑dependently reduced collagen deposition within the pulmonary parenchyma relative to the bleomycin‑treated group (Fig. 8D-F).

## 4 Discussion

Pulmonary fibrosis is a repeatedly encountered adverse effect that may be predisposed by Bleomycin (BLM), an antimicrobial medication utilized as an anticancer drug for the management of many cancers, including ovarian, vaginal, skin, rectal, and cervical cancers [25]. In the current investigation, BLM administration was found to significantly increase BALF LDH activity, total leucocytic count, and the percentage of neutrophils and lymphocytes, and significantly decrease the percentage of macrophages when compared to the control group. This was in agreement with Balaha et al. [26], who reported that BLM produces significant damage to the alveolar epithelial and endothelial cells by generating ROS and inducing DNA strand breaks. This subsequently triggers the inflammatory processes in the pulmonary tissues with entrapment of the circulating leucocytes into the lung [27]. Moreover, et al. stated that the BLM-induced significant elevation of BALF neutrophils is due to the increased production and release of IL-8 and tumor necrosis factor alpha (TNF-α), which attract the neutrophils to the alveoli [28]. BLM-mediated persistent lung injury leads to activation of adaptive immunity, with infiltration of the lung by T-lymphocytes to regulate the inflammatory cascade and induce fibrotic changes [29]. The significant decline in BALF macrophages encountered in the BLM group in the present work was reported to be due to the direct toxic effect of BLM on the macrophages as a consequence of oxidative stress [30]. Additionally, the entrapment of neutrophils and lymphocytes into BALF dilutes the percentage of BALF macrophages [31]. Moreover, some macrophages differentiate into pro-fibrotic phenotypes and migrate deeper into the pulmonary tissues rather than remaining in the airspaces, with a subsequent decline in the percentage of BALF macrophages [32].

The significant increase in the activity of BALF LDH in the BLM group in the current work may originate from the fact that LDH is a key mediator of cellular injury and chemotherapy-induced cytotoxicity in the lung [33]. Tao et al. [34] reported that BLM-induced cell injury leads to loss of the integrity of the cellular membranes with release of the intracellular enzymes like LDH into the extracellular space and BALF. This subsequently drives the repair processes into fibroblast activation and massive collagen deposition into the pulmonary tissues [35].

In the present study, PZ, one of the most distinguished apple polyphenols [36], was shown to attenuate lung fibrosis induced by BLM administration in a dose-dependent manner. PZ-predisposed pulmonary protection was detected by the remarkable decline of elevated BALF total leucocytic count, the percentage of neutrophils and lymphocytes, as well as the notable increase in the percentage of macrophages. In addition, PZ resulted in a significant inhibition of BALF lactate dehydrogenase (LDH) activity. This may be attributed to the potent anti-inflammatory properties of PZ with its ability to inhibit migration of the neutrophils and lymphocytes to the pulmonary tissues; an action that may be mediated via repression of the production of the proinflammatory cytokines [37]. Moreover, PZ was reported to abrogate the direct toxic effects of chemotherapeutic agents on the macrophages and enhance their migration to the inflamed tissues with subsequent modulation of the inflammatory processes [38]. In addition, PZ was reported to combat the deleterious effects of ROS on the cellular integrity of the pulmonary epithelium and endothelium, with a subsequent decrease in LDH activity in BALF [37].

Undoubtedly, oxidative stress is a mainstay pathological pathway critically involved in BLM-predisposed lung fibrosis [39], [40]. In the present study, BLM resulted in a significant elevation of oxidative stress as elucidated by the remarkable increase of MDA and the notable decline of GSH contents. Previous studies correlated between BLM administration and oxidative stress [41]. BLM, as a redox-active compound, generates electron-deficient substances such as hydroxyl radicals, superoxide, and hydrogen peroxide [42]. In the same context, pre-administration of PZ significantly attenuated the increased MDA level and remarkably enhanced GSH level, as compared with BLM-treated rats. Previous investigations have ascertained the antioxidant activity of PZ [43], [44].

Indeed, the pathophysiological role of inflammation in BLM-inflicted lung fibrosis is undeniable [45], [46], [47]. BLM administration predisposes early inflammatory manifestations which are designated by the over-expression of NF-κB, a transcription factor that is mandatory for the production of cytokines which are both pro-inflammatory and Pro-fibrotic [48]. Among the pro-inflammatory cytokines are, IL-1β, IL-6 and TNF-α, followed by elevated levels of pro-fibrotic markers; fibronectin, procollagen-1 and TGF-β1 [49]. In the current study, pre-treatment with PZ remarkably declined elevated IL-1β, NF-κB, and TGF-β1 levels, as compared with BLM-treated rats. Previously, PZ was shown to reduce the inflammatory response in rheumatoid arthritis rats [50]. In addition, PZ guarded against cisplatin-induced nephrotoxicity in mice via suppressing oxidative stress and inflammation [51]. In the same context, PZ protected against carbon tetrachloride-induced hepatic fibrosis in rats as shown by the marked diminution of enhanced mRNA and protein expression of α-smooth muscle actin (α-SMA), TGF-β1and tissue inhibition of metalloproteinase- (TIMP1) [52].

Apoptosis is an undebatable pathological axis that paves the way to BLM-inflicted lung injury. BLM-induced elevation of ROS level leads to the activation of caspase-8, mitochondrial leakage, activation of caspase-9, and finally apoptosis [53], [54]. In the present investigation, PZ was found to dose-dependently down-regulate the enhanced expression of the apoptotic markers, cleaved caspase-3. In a previous study, PZ protected against methotrexate-induced hepatic injury in rats via the attenuation of oxidative stress, inflammation, and apoptosis in hepatic tissues [55].

Autophagy is a well-controlled intracellular degradation machinery through which dysfunctional organelles and proteins are disposed, thus maintaining regular cellular performance [56]. Regarding lung fibrosis, it has been reported that stimulation of pro-fibrotic factor, TGF-β1can inhibit autophagy in lung fibroblasts [57]. Moreover, the autophagic factor, Beclin-1 deficiency, leads to collagen accumulation in the lung fibroblasts [58]. In the present study, PZ was able to dose-dependently upregulate the expression of the autophagic factor, beclin-1, as compared with the BLM group. Previous research has shown that PZ improved cardiovascular injury via autophagic activation [59].

While the present study demonstrates that PZ can attenuate BLM-induced pulmonary fibrosis in male Wistar rats, several limitations should be considered. The findings are restricted to an experimental animal model, which may not fully capture the complexity and heterogeneity of human pulmonary fibrosis, thereby limiting direct clinical applicability. The BLM model itself represents an acute injury pattern and doesn't encompass all aspects of chronic pulmonary fibrotic disease seen in most patients. Moreover, this study was conducted in a single sex and species, raising questions about the potential generalizability across different biological contexts. Also, the present study didn’t investigate whether PZ affects the antitumor activity of BLM.

## 5 Conclusion

PZ ameliorated BLM-induced pulmonary fibrosis through inhibition of oxidative stress, inflammation, apoptosis, and fibrosis, along with upregulation of autophagy. This may open new gates towards the attenuation of BLM-elicited pulmonary toxicity. Future investigations using alternative models of fibrosis, larger animal groups, longer treatment durations, and ultimately clinical studies will be essential to validate the therapeutic potential of PZ in this context and to establish its clinical relevance in human disease. Also, exploration of the impact of PZ on the antitumor effects of BLM in cancer-bearing animal models should be considered.

## CRediT authorship contribution statement

**Ahmed M. Kabel:** Writing – review & editing, Visualization, Validation, Methodology, Investigation, Formal analysis. **Amany A.E. Ahmed:** Writing – review & editing, Visualization, Validation, Methodology, Investigation, Formal analysis, Conceptualization. **Naif M.F. Alenezi:** Writing – review & editing, Writing – original draft, Visualization, Validation, Methodology, Investigation, Formal analysis, Data curation. **Abeer Elkhoely:** Writing – review & editing, Visualization, Validation, Methodology, Investigation, Formal analysis, Conceptualization.

## Informed consent

Not applicable.

## Ethics approval

All the experimental methods were conducted in Medical Pharmacology Department of the Faculty of Medicine, Tanta University, Egypt, and Pharmacology and Toxicology Department of the Faculty of Pharmacy, Helwan University, Egypt. Animal handling and experimental procedures adhered to the rules established by the Research Ethics Committee of the Faculty of Pharmacy, Helwan University, Egypt (Approval code 15A2024).

## Funding

This research didn’t receive any funding.

## Declaration of Competing Interest

The authors declare that they have no known competing financial interests or personal relationships that could have appeared to influence the work reported in this paper.

## Data Availability

Data will be made available on request.
